# Effect of Storage Time and Temperature on Digestibility, Thermal, and Rheological Properties of Retrograded Rice

**DOI:** 10.3390/gels9020142

**Published:** 2023-02-08

**Authors:** Ishita Chakraborty, Indira Govindaraju, Steffi Kunnel, Vishwanath Managuli, Nirmal Mazumder

**Affiliations:** 1 Department of Biophysics, Manipal School of Life Sciences, Manipal Academy of Higher Education, Manipal 576104, Karnataka, India; 2 Department of Mechanical and Industrial Engineering, Manipal Institute of Technology, Manipal Academy of Higher Education, Manipal 576104, Karnataka, India

**Keywords:** rheology, resistant starch, gelatinization, retrogradation

## Abstract

Retrogradation is defined as the recrystallization or realignment of amylose and amylopectin chains upon cooling of gelatinization starch gels. The storage conditions such as the storage time and temperature are crucial factors that influence and govern the degree of retrogradation and in turn, affect the formation of resistant starch and alteration of thermal and rheological properties. This article investigates the effect of storage time and temperature on the properties of retrograded rice starch. Rice kernels of five different indigenous varieties, namely Diasang lahi, Khaju lahi, Dhusuri bao, Omkar, and Bili rajamudi were cooked by boiling in water and stored at 4 °C and −20 °C for 6 and 12 h, respectively. Differential scanning calorimetry (DSC) studies revealed in raw form that Bili rajamudi exhibited the highest peak gelatinization temperature (T_p_, °C) at 79.05 °C whereas Diasang lahi showed the least T_p_ at 56.12 °C. Further, it was indicated that the T_p_ and degree of retrogradation (DR%) also increase with increasing time and decreasing temperature of storage. All samples stored at −20 °C for 12 h exhibited the highest degree of retrogradation DR%. Amongst all five varieties stored at −20 °C for 12 h, Omkar exhibited the highest %DR, followed by Bili rajamudi, Khaju lahi, Dhusuri bao, and Diasang lahi. A negative correlation was also established between T_p_ and resistant starch content (RS%). It was also observed that the resistant starch (RS%) content increased with the increasing time and decreasing temperature of storage. A strong negative correlation was observed between RS% and non-resistant starch (NRS%). Further, rheological studies indicated that retrogradation also affects the viscosity and dynamic rheological properties of starch. In this study, it was evident that extending storage duration from 6 to 12 h and lowering temperature from 4 to −20 °C impact retrogradation of rice starch, which in turn affects the starch’s gelatinization, digestibility, and rheology. Rice starch retrograded at lower temperatures for a longer period could prove to be extremely beneficial for development of food products with better textural properties and high RS content or low glycemic index.

## 1. Introduction

Rice is one of the most economically indispensable crops, that contributes to the staple diet for most of the world’s population. The quality of rice-based food products essentially depends on the properties of its main component, starch [1]. Starch is the ultimate storage carbohydrate in plants and is further composed of linear amylose and branched amylopectin. Starch is used broadly in food industries because of its abundance, renewability, and low price [2]. Gelatinization and retrogradation are essential phenomena that occur in starch-based food products. It is vital to recognize the starch behavior during gelatinization and retrogradation to obtain the food products with desired rheological and textural characteristics, and digestibility. However, various factors such as amylose content, storage temperature, time, and moisture content could lead to varying characteristics of the final product [3]. 

Starch is usually gelatinized by heating in the presence of water, which results in the disruption of the native starch granules along with an order-to-disorder transition [4]. On storage or cooling after gelatinization, the disordered amylose and amylopectin chains gradually reassociate or rearrange into a different ordered structure, and this is known as retrogradation [5]. Starch retrogradation is accompanied by changes in several physical and functional properties such as texture, flavour, viscosity, and digestibility. 

The time and temperature of storage are major factors affecting the process of starch retrogradation [5]. Based on its digestibility, Total starch (TS) is categorized into resistant starch (RS), and non-resistant starch (NRS). RS is characterized as a starch fraction that does not undergo digestion or breakdown by digestive enzymes in the human gastrointestinal tract and hence results in a low glycemic index. It has been reported that retrogradation of starch alters its RS content and ultimately varies its digestibility. On the other hand, NRS is the starch faction that is easily digested and releases glucose into the bloodstream after consumption [6]. A study that reported that in waxy rice starch, retrogradation resulted in decreased RS content (RS%). Further, it was noted that with increasing time of storage at 4 °C (2, 4, and 7 days), the RS% increased [7]. The thermal properties of retrograded rice gels stored for 1, 3, and 6 days using differential scanning calorimetry (DSC). All the samples showed single endothermal peaks between 35.5 and 69.0 °C, with different enthalpies accredited to different storage times. It was found that differential scanning calorimetry (DSC) endothermal peaks of recrystallized starch gel samples occurred at considerably lower temperatures than those of the original gelatinized samples [8].

In addition to alterations in thermal properties and RS%, retrogradation also affects the rheological properties of starch. Rheological characterization of starch includes evaluation of its flow and deformation. The rheological property of starch impacts its important applications as thickeners and binders in the food and pharmaceutical industries. Starch rheology plays a crucial role in the regulation, production, optimization, and sensory properties of starch-based food products. Rheological properties also indicate the behavior of starch as the raw materials and quality end products after processing. This study attempts to investigate the effect of time and temperature of storage on the digestibility, thermal, and rheological properties of five different varieties retrograded rice collected from the states of Assam and Karnataka, India. These rice varieties were chosen based on their apparent amylose content (AAC%). 

## 2. Results and Discussion

### 2.1. Determination of Thermal Properties

**Figure 1:**
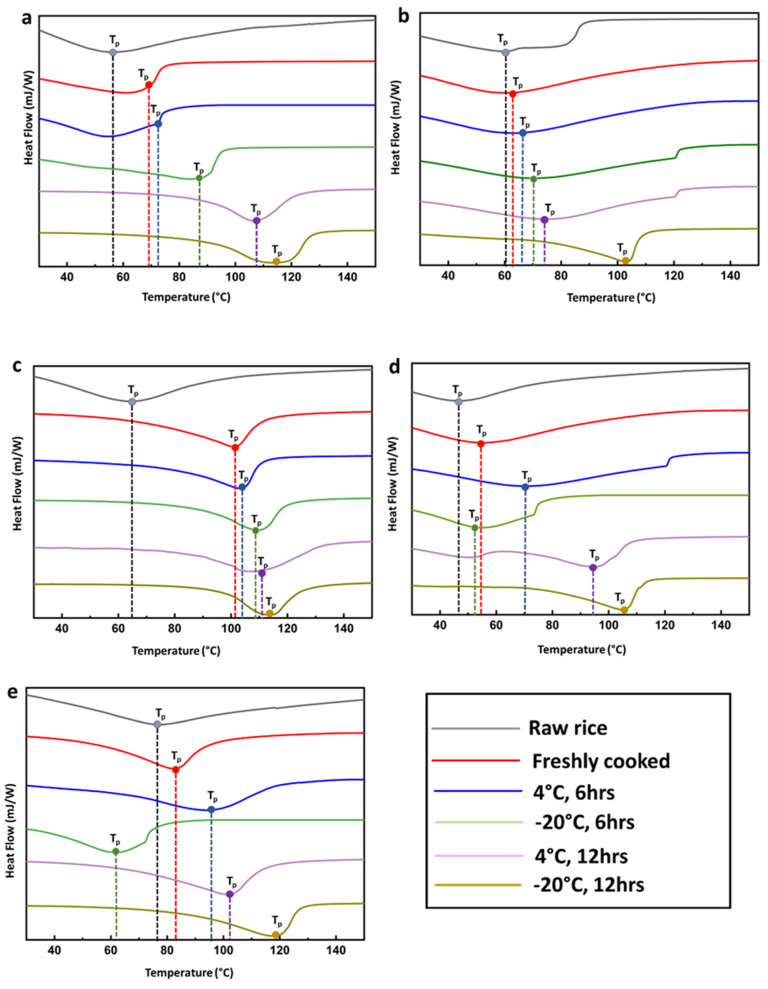
DSC endotherms showing peak gelatinization (T_p_) of native starch and retrograded rice samples: (**a**) Diasang lahi, (**b**) Khaju lahi, (**c**) Dhusuri bao, (**d**) Omkar, and (**e**) Bili rajamudi.

Starch starts to retrograde and form a crystalline structure immediately as the cooked or gelatinized starch begins to cool down. Figure 1 shows a characteristic endothermic peak indicating the gelatinization of starch present in the rice kernels. Retrogradation occurs when the starch constituent’s amylose and amylopectin molecules begin to reassociate into an ordered structure [9]. It is observed that, as a general trend, the peak gelatinization temperature (T_p_) increases as the time of storage increases (Figure 1). For all five varieties, T_p_ is the lowest for raw rice. The T_p_ increases for freshly cooked rice followed by cooked rice stored for 6 and 12 h, respectively. Bili rajamudi exhibits an exception, where reheating after being stored at 4 °C for 6 h shows a lower T_p_ compared to the raw rice. This may be accredited to the presence of sufficient moisture leading to premature gelatinization of starch granules. Further, the peak becomes larger and more intense with increasing time of storage at 12 h. Since the size of this peak implies progress in the degree of retrogradation in stored or refrigerated rice, it is assumed that retrogradation increases with time. 

Further, it can also be seen that as the temperature of storage decreases from room temperature to 4 °C, and −20 °C, the T_p_ increases. This is because the strong bonds between amylose chains during retrogradation cause more water to expel from the starch gel (syneresis) when it is subjected to lower temperatures [10,11]. Hence, starch retrograded at 4 °C shows a lower %DR than those stored at −20 °C at both 6 and 12 h of storage. Further, lower temperatures result in reduced starch swelling (supplementary materials, Figure S3) which delays gelatinization [12]. Table 1 shows the T_p_ of native starch and retrograded rice samples. Since amylose is more susceptible to retrogradation than amylopectin, the influence of retrogradation on gelatinization temperatures is less pronounced for varieties belonging to the waxy group (exhibiting AAC% ≤ 10%). The very low amylose level makes the structural retrogradation less noticeable.

**Figure 2:**
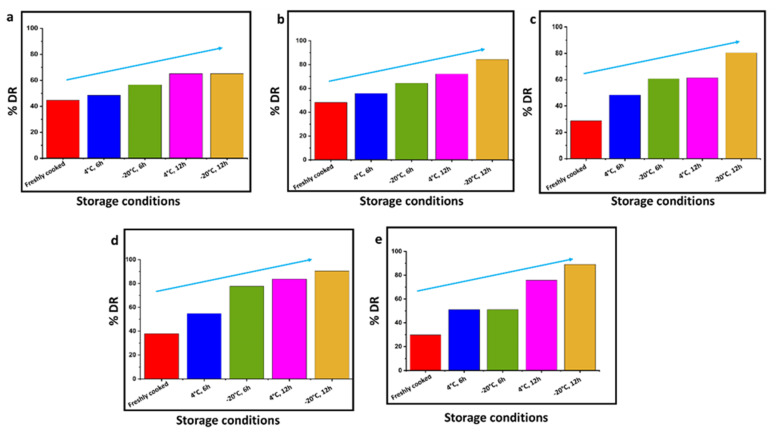
Degree of retrogradation (%DR) increases with increasing time and decreasing temperature of storage: (**a**) Diasang lahi, (**b**) Khaju lahi, (**c**) Dhusuri bao, (**d**) Omkar, and (**e**) Bili rajamudi.

Table 2 shows the gelatinization enthalpy change of native starch (ΔH_G_) and enthalpy change on reheating of retrograded starch (ΔH_R_). ΔH_G_ and ΔH_R_ indicate the starch crystallinity which in turn indicates the loss of molecular order within the starch granule subjected to gelatinization [13]. It was observed that ΔH_G_ was highest for raw rice. For the retrograded rice, ΔH_R_ was lower for the freshly cooked rice compared to the ΔH_G_ of raw rice. Further, with increasing time and decreasing temperature of storage, the ΔH_R_ for stored rice was higher than the freshly cooked rice at room temperature (Table 2). Based on the ΔH_G_ and ΔH_R_, the degree of retrogradation (%DR) of rice samples with increasing time and decreasing temperature of storage was calculated and depicted in Figure 2 (blue arrow indicates the increase in %DR). The %DR is affected by several parameters such as starch granule size, amylose content, starch concentration, moisture content, and storage conditions [14]. As expected, freshly cooked rice at room temperature exhibited the least DR% as opposed to rice stored at −20 °C for 12 h. A study reported that the endothermal peak obtained from reheating retrograded amylopectin exhibited a peak between 50 and 80 °C. It was anticipated that during retrogradation the amorphous region of gelatinized starch is transformed into a crystalline one, resulting in a different crystalline-amorphous ratio compared to the native structure [15]. 

However, as the time of storage increased from 6 h to 12 h, the gelatinized starch molecules tended to rearrange and form more ordered and stable structures leading to a higher %DR. According to a recent study, short-term retrogradation of starch often takes place within a few hours and is completely irreversible due to amylose recrystallization. However, long-term retrogradation begins after 12 h of storage and is reversible. This retrogradation is driven by amylopectin recrystallization. [16].

In a similar study, it was reported that the peak temperature of retrograded amylose occurs between 100 to 165 °C [17]. In another similar study, the thermal characteristics of retrograded rice, and wheat starch were studied using DSC. It was observed that rice and wheat starches displayed a typical endothermic gelatinization pattern with transition temperatures temperature ranging between 61.2–73.9 °C and 55.1–67.4  °C, respectively. A much broader and shallower endothermic peak was obtained ranging between 42.9–68.9  °C and 41.9–65.3  °C retrograded rice and wheat starch, respectively [18]. The area under the retrogradation endotherms increased at first and then decreased as storage time increased. This is again accredited to the structural rearrangement of amylose and amylopectin chains within the starch granule during retrogradation. 

The alteration in the thermal properties of retrograded rice compared to native raw rice starch is beneficial for several applications in the food industry such as rice vermicelli, noodles, breakfast cereals, and parboiled rice. 

### 2.2. Determination of Total Starch, Non-Resistant Starch, and Resistant Starch Content

**Figure 3:**
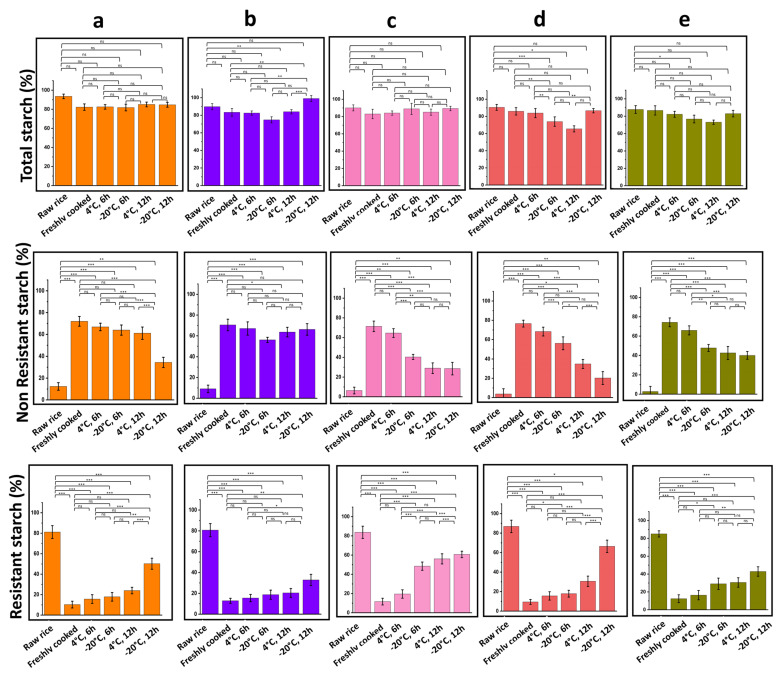
Total starch (TS%), non-resistant starch (NRS%), and resistant starch (RS%) with increasing time and decreasing temperature of storage: (**a**) Diasang lahi, (**b**) Khaju lahi, (**c**) Dhusuri bao, (**d**) Omkar, and (**e**) Bili rajamudi. (^ns^ *p* > 0.05, * *p* < 0.05, ** *p* < 0.01, *** *p* < 0.001).

Determination of TS%, NRS%, and RS% of raw, freshly cooked, and retrograded rice varieties are depicted in Figure 3. It was observed that the total starch content (TS%) does not vary much amongst raw, freshly cooked, and retrograded starch samples. Slight variations may occur due to the cooking of rice in excess water. The starch granules in rice when heated in water, swell and burst, leading to loss of starch content in freshly cooked rice [19]. In freshly cooked rice, a rapid fall in the resistant starch (RS%) content and a rise in NRS% are observed. It has already been established that cooling of freshly cooked rice leads to starch retrogradation which in turn increases RS% by recrystallization of some single chains (amylose) to form double helices (amylopectin) via strong hydrogen bonds leading to increased crystallinity and RS%. In raw rice, the RS is classified as type II, where intact granules cannot be gelatinized due to the compact structure and thus cannot be digested by enzymes. Upon retrogradation type I, RS is converted into type III RS. Formation of type III RS is common a common phenomenon that occurs during food processing. Further, it has been previously reported that retrogradation is favored by amylose more than with amylopectin since it is a smaller unbranched, and linear molecule. Hence, waxy starches with minimal amylose content are often favored to reduce the side effects of retrogradation in foods. It is observed that for each rice variety, the RS% is highest in raw rice [20]. The RS% is decreased for freshly cooked rice and increases with increasing time storage. Rice stored for 12 h displays higher content of RS% compared to the rice stored for 6 h. 

Further, it is also observed that rice stored at −20 °C shows more RS% compared to rice stored at 4 °C. This is because at 4 °C, retrogradation occurs spontaneously as the cooked rice begins to cool, reducing its digestibility as indicated in by the increasing RS% (Figure 3, Table 3). In addition, at freezing temperatures of −20 °C, the formation of ice crystals occurs followed by syneresis of water from the starch gel adds to the formation of RS in retrograded rice. The DR% has a significant impact on how RS forms in cooked starches held at refrigerated temperatures. RS% in turn affects its digestibility and estimated glycaemic index. The benefits of increased RS% content in retrograded and refrigerated rice also include better gut health, and lower cholesterol levels [21]. 

In a similar study, the digestibility of six indigenous Philippine rice cultivars subjected to refrigeration at 4 °C for 24 h was studied. The results indicated substantial differences in the estimated glycaemic index between the rice cultivars [22]. For cooked rice and rice that had been refrigerated, the glycaemic index values varied from 68 to 109 and 64 to 87, respectively [22]. In a study conducted, [23] examined how retrogradation affected the amount of resistant starch in three different types of cooked white rice: control rice, test rice I, and rice cooled after 24 h at 4 °C (test rice II). The results showed that resistant starch contents were higher in test rice II compared to control rice, and test rice I. Control rice, and test rice I test rice II were 0.64%, 1.30%, and 1.65%, respectively [23].

### 2.3. Determination of Apparent Viscosity and Dynamic Rheological Properties of Rice

**Figure 4:**
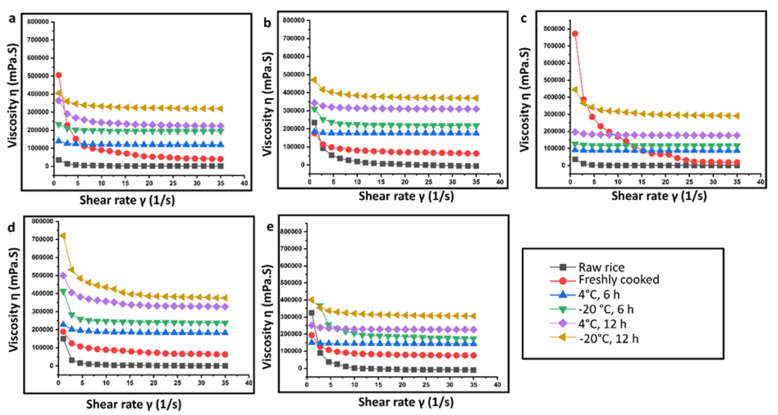
Relationship between apparent viscosity and shearing rate of rice samples subjected to different storage conditions for retrogradation: (**a**) Diasang lahi, (**b**) Khaju lahi, (**c**) Dhusuri bao, (**d**) Omkar, and (**e**) Bili rajamudi.

Rice starch with favorable rheological properties is used in a variety of food products with distinct properties. The relationship between apparent viscosity and shearing rate of rice samples subjected to different storage conditions for retrogradation is exhibited in Figure 4. It was observed that, in general, as the shear rate increased, the apparent viscosity of all the rice gels decreased indicating shear thinning property (thixotropy). Rice starch gels form a stable hydrogen bond network [24]. In addition, increasing the shear rate damages the starch gel network and results in a decrease in apparent viscosity. Further, it is reported that RS exhibits low water binding properties. Hence, with an increase in the RS%, the apparent viscosity decreases. In addition, reduced water absorption also results in delayed swelling, and an increased T_p_ (Table 1). As observed in the previous section, the RS% increases in refrigerated rice samples stored at 4 and −20 °C for 6 and 12 h. Studies report that viscosity of food gets affected by water availability during starch hydrolysis and in turn affects the absorption of carbohydrates by the digestive tract [25]. 

**Figure 5:**
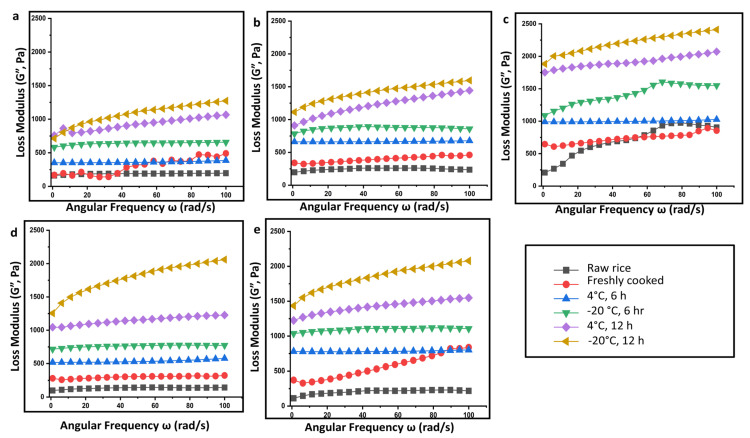
Loss modulus (G”) as a function of the angular frequency of rice samples subjected to different storage conditions for retrogradation: (**a**) Diasang lahi, (**b**) Khaju lahi, (**c**) Dhusuri bao, (**d**) Omkar, and (**e**) Bili rajamudi.

**Figure 6:**
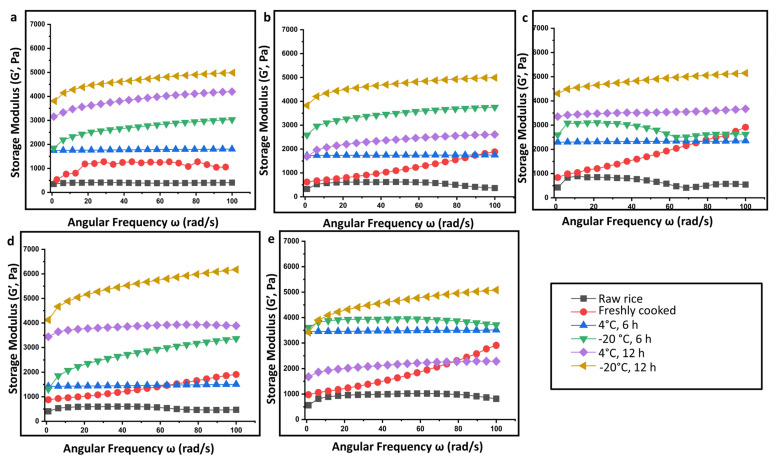
Storage modulus (G’) as a function of the angular frequency of rice samples subjected to different storage conditions for retrogradation: (**a**) Diasang lahi, (**b**) Khaju lahi, (**c**) Dhusuri bao, (**d**) Omkar, and (**e**) Bili rajamudi.

Compared to the raw rice, the overall apparent viscosity values of retrograded rice increased markedly. Further, the viscosity is also seen to increase with a decrease in temperature from 4 °C to −20 °C. At lower temperatures, the starch molecules have low thermal energy. This inhibits them from overcoming the attractive forces (hydrogen bonds) binding them together [26]. With the increment of retrogradation time from 6 h to 12 h, the apparent viscosity values continuously increase during retrogradation to be more shear-resistant and shear-stabilized. It was reported that an increase in viscosity can impair gastric function and disrupts the peristalsis during in vivo digestion [27]. The dynamic rheological properties of rice starch gel from different rice cultivars were determined in terms of storage modulus, loss modulus, and loss factor as a function of angular frequency. The initial increase in G’ and G” (Figure 5 and Figure 6) could be attributed to the granular swelling followed by a decrease in G’ and G”, indicating that the gel structure is destroyed as the angular frequency increases. Increasing angular frequency results in the destruction of the crystalline region of the retrograded starch granules. 

It was reported that the G’ and G” values were higher for non-waxy rice and corn starches than the waxy corn and rice starches. Additionally, they observed that the addition of amylose to waxy rice and corn starches resulted in a rise in G’. Waxy corn and rice cultivars’ starches have lower G’ and G” than regular corn and rice types. Unlike regular starch, which has starch granules embedded in a continuous network of amylose, waxy starches experience the phenomena of gel formation [28].

In a study [29], dynamic shear rheological tests at 4 °C were conducted, indicating that octenyl succinic anhydride (OSA) modified potato starch exhibited a feeble gel-like behavior with the storage moduli (G’) higher than the loss moduli (G”) over most of the frequency ranges (0.63–63.8 rad/s) [29]. 

**Figure 7:**
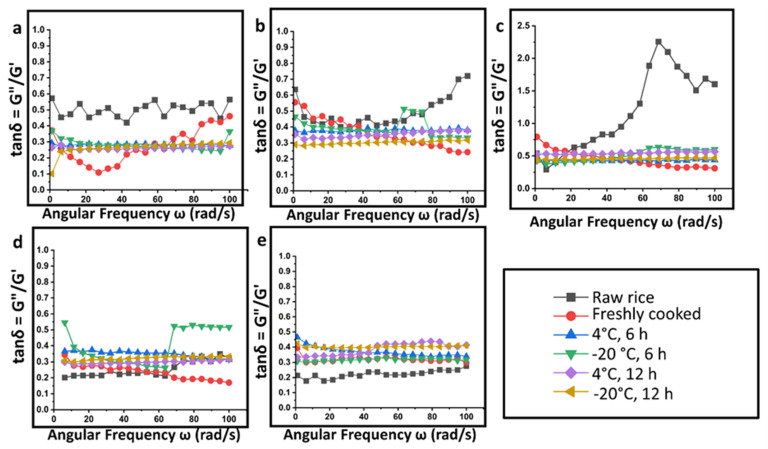
Loss tangent (tanδ = G”/G’) as a function of the angular frequency of rice samples subjected to different storage conditions for retrogradation: (**a**) Diasang lahi, (**b**) Khaju lahi, (**c**) Dhusuri bao, (**d**) Omkar, and (**e**) Bili rajamudi.

In general, the tan δ values of the retrograded rice samples were lower than one, indicating the samples have a typical gel network (Figure 7). Lower tan δ values indicate stronger and more gel-like pastes. Further, the lower tan δ values and prominent gel-like behaviour in rice samples stored at freezing temperatures is attributed to the increasing RS%. It is evident that retrograded rice samples exhibit lower tan δ values compared to the raw rice samples. The rheological properties of rice starch determine its applications as gelling agents or thickeners in the food industry. 

### 2.4. Correlation Matrix

**Figure 8:**
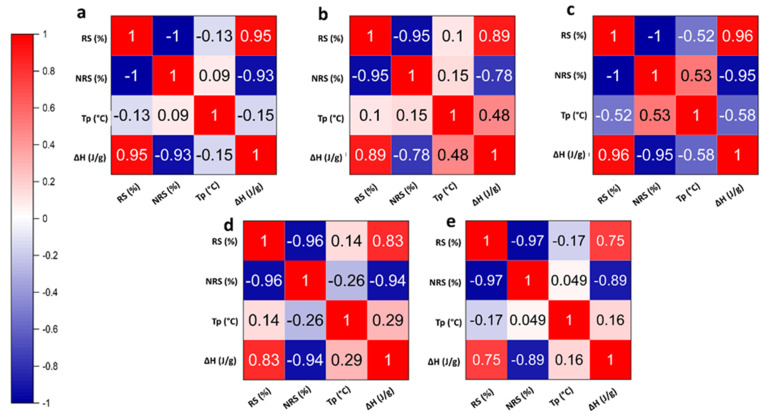
Matrix showing correlation between resistant starch content (RS%), non-resistant starch (NRS %), peak gelatinization temperature (Tp), and gelatinization/retrogradation enthalpy (ΔH): (**a**) Diasang lahi, (**b**) Khaju lahi, (**c**) Dhusuri bao, (**d**) Omkar, and (**e**) Bili rajamudi.

A correlation matrix was plotted to analyze the relationship between RS%, NRS%, Tp, and ΔH. A similar trend was observed for all 5 samples, as exhibited in Figure 8. It was observed that RS% and NRS% exhibit a strong negative correlation and are complimentary to each other. This indicates that during retrogradation, the NRS% converts into RS% due to the rearrangement of the amylose and amylopectin chains. Further, there is a positive correlation between ΔH and RS%. This may be attributed to increased hydrogen bond formation and improved starch stability between long-chain branches. In addition, Diasang lahi, Dhusuri bao, and Bilirajamudi show a weak negative correlation was observed between T_p_ and RS% as opposed to Khaju lahi and Omkar. Based on the correlation coefficients, it may be suggested that no linear relationship exists between Tp and RS% or NRS%. 

## 3. Conclusions

This study provides useful information on how to monitor the retrogradation of starchy foods using RS%, thermal, and rheological studies. Retrogradation has a significant impact on the activities of the rice starch food business. Retrogradation is recognized to have an impact on the staleness and hardness of cooked rice. However, the current study emphasises the advantageous uses of retrogradation that may be found in a variety of rice-starch based food products, such as ready-to-eat rice products, breakfast cereals, and rice noodles/vermicelli. The current study also highlights the impact of retrogradation temperature and time on functional qualities such rheological parameters and RS%. Retrograde rice starch is, therefore, preferred for the manufacture of food products with improved texture and greater RS%. The results from this study indicate that the practical application of retrograded starch is found to be more prominently at lower temperatures and with longer storage time. Further, rice varieties with intermediate or high AAC% such as Khaju lahi are more prone to retrogradation as they show the highest %DR and can be easily used to reap the benefits of retrograded starch for various applications. RS% produced from retrograded rice starch and T_p_ is highest for cooked rice stored at −20 °C for 12 h. Amongst samples stored at −20 °C for 12 h, Omkar exhibited the highest RS%, followed by Diasang lahi, Bili rajamudi, Khaju lahi, and Dhusuri bao. However, at −20 °C for 12 h, Bili rajamudi had the highest T_p_ followed by Dhusuri bao, Diasang lahi, Omkar, and Khaju lahi. Further, it was evident that all five varieties exhibited increased RS% with increased DR%. Investigating the viscosity and dynamic rheological properties of starch and starch-based foods gives a direct assessment of their processability. Food rheology contributes to giving crucial information for process design, quality assurance, sensory evaluation, and product development. It was evident that with increasing time and decreasing temperature of storage, both G’ and G” increased, whereas the tan δ was seen to be decreasing. These findings indicate that retrogradation can be used to improve the quality of rice starch-based food products by producing a higher amount of beneficial resistant starch, increasing the T_p_, and improving the rheology properties. In many Asian nations, rice is a common food. For diabetic patients, it has long been thought that consumption of stale or stored rice is not preferable compared to recently prepared rice. However, the present study contradicts this idea. Due to retrogradation, some of the cooked rice’s starch becomes resistant to digestion, which prevents it from being absorbed in the small intestine. In contrast to freshly cooked rice, stale or stored rice may exhibit a decreased glycaemic response based on the increased RS%. Further, the hindrance of gelatinization also hinders the availability of starch for amylase hydrolysis. Hence, stored cooked rice is a better option for diabetic patients.

## 4. Materials and Methods

### 4.1. Materials

Five indigenous varieties, namely Diasang lahi, Khaju lahi, Dhusuri bao, Omkar, and Bili rajamudi were collected from the paddy fields during harvest season. Diasang lahi, Khaju lahi, Dhusuri bao were collected from the state of Assam, India, whereas Omkar, and Bili rajamudi were collected from the fields of Karnataka, India. The seeds were threshed, sun-dried, de-husked, and milled using a McGill Mill No. 2. The kernels were stored at −80 °C for further experimentation [30]. The apparent amylose content (AAC%) of all the five varieties were estimated using iodine binding assay. The detailed methodology and results are mentioned in Supplementary information, Figure S1. Khaju lahi exhibited the highest AAC% (23.56%), followed by Diasang lahi (17.34%), Omkar (15.49%), Dhusuri bao (8.29%), and lastly Bili rajamudi (6.38%). Based on the AAC%, Dhusuri bao and Bili rajamudi were classified as waxy (<10% AAC), Diasang lahi, and Omkar were classified as low (10–20% AAC), and Khaju lahi was classified as intermediate (20–25% AAC). 

### 4.2. Preparation of Retrograded Rice

Initially, the rice was cooked in excess water at the minimum cooking temperature (MCT) determined previously (Supplementary materials, Figure S2). The freshly cooked rice kernels were strained to remove any additional moisture and stored in the freezer for retrogradation at 4 and −20 °C for 6 and 12 h, respectively. 

### 4.3. Determination of Thermal Properties of Rice

Differential scanning calorimetry (DSC) (Shimadzu’s DSC60, Japan) was used to investigate the gelatinization and retrogradation properties of various rice varieties. A total of 3 mg of raw rice, freshly cooked rice, and stored or retrograded rice kernels were weighed into an aluminium pan along with distilled water to obtain a ratio of 1:1 (w/w), sealed, and heated from 30 °C to 160 °C. The experiment was conducted once for each sample. The thermograms were analyzed using TA−60 (Shimadzu, Japan) software. The degree of retrogradation (%DR) was calculated based on the following formula:

(1) $$\;\%\mathrm{DR}=\frac{{\Delta H}_{R}}{{\Delta H}_{G}}\times 100$$

where as the gelatinization enthalpy changes for native starch (ΔH_G_) and there was an enthalpy change on reheating of retrograded starch (ΔH_R_) [31].

### 4.4. Determination of Total Starch, Non-Resistant Starch, and Resistant Starch Content of Rice

Using the RS assay kit, the resistant starch (RS) content of rice samples was calculated (Megazyme International Ireland Ltd., Bray, Ireland). Briefly, a 100 mg sample was digested in 4 mL of pancreatic amylase (10 mg/mL) with amyloglucosidase (300 U/mL) at 37 °C with continuous agitation at 200 rpm for 16 h, and then 4 mL of 100% ethanol was added with vigorous stirring. Next, centrifugation at 3000 rpm for 10 min followed. The pellet was centrifuged at 3000 rpm for 10 min while suspended in 8 mL of 50% ethanol. The non-resistant starch content of the supernatant was analysed. The pellet was resuspended in 2 M KOH for 20 min, and then 8 mL of 1.2 M sodium acetate buffer and 0.1 mL of amyloglucosidase (3300 U/mL) were added. The tubes were centrifuged at 3000 rpm for 10 min after being incubated at 50 °C for 30 min. A different set of test tubes received aliquots of 0.1 mL of supernatant, to which 3 mL of glucose oxidase/peroxidase (GOPOD) reagent was added. For 20 min, these tubes were incubated at 50 °C. At 510 nm, there was a comparison to a blank for the reagent. Based on the readings, the RS%, NRS%, and TS% were determined [32].

### 4.5. Determination of Apparent Viscosity and Dynamic Rheological Properties of Rice

Briefly, using a mortar and pestle, the rice kernels were mashed into a smooth paste and immediately transferred to a parallel plate rheometer (Anton Paar, Austria) with a diameter of 50 m diameter, and a gap size of 1 mm. The continuous shear tests were carried out at 37 °C, over a shear rate range of 0–40 s^−1^, to determine the effect of shear rate on apparent viscosity and to describe the steady shear rheological properties of the samples, and the data was fitted to the well-known Herschel–Bulkley power law model.

For the study of the dynamic rheological properties, frequency sweeps were performed at 37 °C, over the angular frequency range of 0–100 rad/s at 2% strain. The mechanical spectra were obtained to record the storage modulus (G’), loss modulus (G”), and loss tangent (tanδ = G”/G’) as a function of the frequency (ω). [33]. 

### 4.6. Statistical Analysis

The mean and standard deviation of values from the resistant starch content (RS%), non-resistant starch (NRS%), and total starch (TS%) content were obtained in triplicate using a built-in function in Microsoft Excel 2013. A one-way analysis of variance (ANOVA) was used to evaluate the differences among the means of obtained values for TS%, NRS%, and RS% using Graphpad Prism 9 (Learning edition). A level of *p*  <  0.05 was used followed by Tukey’s significant difference test to compare the means of each case. Pearson correlation among the RS%, NRS%, Tp, and ΔH_G/R_ was calculated using OriginPro2022b (Learning Edition).

## Figures and Tables

**Table 1:** Peak temperature of gelatinization (T_p_, °C) of native starch and retrograded rice samples.

Sample	Peak Temperature of Gelatinization (T_p_, °C)					
	Raw Rice	Freshly Cooked (Room Temperature)	6 h Stored		12 h Stored	
			4 °C	−20 °C	4 °C	−20 °C
Diasang lahi	56.12	61.21	72.81	84.10	106.88	109.75
Khaju lahi	58.53	62.41	64.72	69.73	74.09	100.40
Dhusuri bao	64.55	101.28	102.65	106.88	106.90	110.74
Omkar	46.82	52.26	69.73	57.05	92.37	107.82
Bili rajamudi	79.05	84.25	93.78	61.46	101.28	119.75

**Table 2:** Gelatinization enthalpy change of native starch (ΔH_G_) and enthalpy change on reheating of retrograded starch (ΔH_R_) for rice samples.

Sample	Raw Rice	Freshly Cooked	6 h Stored		12 h Stored	
	ΔH_G_	ΔH_R_	ΔH_R_ (4 °C)	ΔH_R_ (−20 °C)	ΔH_R_ (4 °C)	ΔH_R_ (−20 °C)
Diasang lahi	23.49	10.48	11.39	13.29	15.29	15.29
Khaju lahi	25.48	12.28	14.2	16.39	18.37	21.47
Dhusuri bao	15.29	4.39	7.39	9.27	9.38	12.29
Omkar	13.49	5.1	7.39	10.47	11.29	12.2
Bili rajamudi	8.3	2.48	4.24	6.29	7.26	7.39

**Table 3:** Total starch% (TS%), Non-resistant starch% (NRS%), and Resistant starch% (RS%) of rice varieties.

	Sample	Raw Rice	Freshly Cooked	6 h Stored		12 h Stored	
				4 °C	−20 °C	4 °C	−20 °C
Total starch (TS%)	Diasang lahi	93.52 ± 2.37 ^a^	82.26 ± 3.39 ^a^	82.52 ± 2.39 ^a^	81.81 ± 3.48 ^a^	85.05 ± 2.49 ^a^	84.67 ± 2.50 ^a^
	Khaju lahi	89.769 ± 3.49 ^a^	83.33 ± 4.49 ^a^	82.50 ± 2.43 ^a^	74.77 ± 3.49 ^a^	84.00 ± 2.49 ^b^	99.02 ± 3.48 ^c^
	Dhusuri bao	90.0636 ± 3.48 ^a^	82.98 ± 5.48 ^a^	84.07 ± 2.48 ^a^	88.89 ± 6.48 ^a^	85.13 ± 3.56 ^a^	89.34 ± 2.58 ^a^
	Omkar	90.55 ± 3.45 ^a^	85.95 ± 4.34 ^a^	83.86 ± 5.34 ^a^	73.95 ± 5.48 ^b^	65.47 ± 3.56 ^c^	86.67 ± 2.56 ^a^
	Bili rajamudi	87.75 ± 4.48 ^a^	86.66 ± 5.36 ^a^	82.26 ± 3.45 ^a^	76.78 ± 4.46 ^b^	73.13 ± 2.45 ^a^	82.98 ± 3.78 ^a^
Non- resistant starch (NRS%)	Diasang lahi	12.35 ± 3.47 ^a^	71.99 ± 4.47 ^b^	66.85 ± 3.45 ^b^	63.96 ± 4.67 ^b^	61.09 ± 5.67 ^b^	34.45 ± 4.69 ^b^
	Khaju lahi	9.06 ± 3.58 ^a^	70.49 ± 3.58 ^b^	67.05 ± 3.58 ^b^	56.09 ± 3.58 ^b^	63.62 ± 3.58 ^b^	66.16 ± 3.58 ^b^
	Dhusuri bao	6.36 ± 3.4 ^a^	71.36 ± 5.34 ^b^	64.55 ± 4.45 ^b^	40.41 ± 2.48 ^c^	29.04 ± 5.4 ^c^	28.62 ± 6.39 ^c^
	Omkar	3.69 ± 1.45 ^a^	76.55 ± 3.56 ^b^	68.27 ± 4.57 ^b^	56.09 ± 6.67 ^c^	34.85 ± 4.68 ^b^	20.25 ± 6.68 ^c^
	Bili rajamudi	2.72 ± 1.35 ^a^	74.24 ± 4.56 ^b^	65.89 ± 4.68 ^b^	47.68 ± 3.59 ^c^	42.51 ± 6.69 ^c^	40.08 ± 3.9 ^c^
Resistant starch (RS%)	Diasang lahi	81.17 ± 6.29 ^a^	10.26 ± 3.29 ^b^	15.66 ± 4.29 ^b^	17.85 ± 4.2 ^b^	23.96 ± 3.2 ^b^	50.22 ± 5.4 ^c^
	Khaju lahi	80.69 ± 6.3 ^a^	12.84 ± 2.4 ^b^	15.44 ± 3.49 ^b^	18.68 ± 4.39 ^b^	20.37 ± 4.3 ^b^	32.85 ± 5.39 ^c^
	Dhusuri bao	83.69 ± 6.39 ^a^	11.61 ± 3.49 ^b^	19.51 ± 4.38 ^b^	48.48 ± 4.29 ^c^	56.09 ± 5.29 ^c^	17.71 ± 3.38 ^b^
	Omkar	86.86 ± 6.39 ^a^	9.39 ± 2.49 ^b^	15.59 ± 4.29 ^b^	17.86 ± 3.49 ^b^	30.62 ± 5.29 ^b^	66.41 ± 6.39 ^c^
	Bili rajamudi	85.02 ± 3.48 ^a^	12.41 ± 4.39 ^b^	16.37 ± 5.24 ^b^	29.10 ± 6.39 ^b^	30.62 ± 5.29 ^b^	42.89 ± 5.29 ^c^

In a row, the same letters in superscript indicate no statistical significance.

## Data Availability

The data presented in this study are available on request from the corresponding author.

## Supplementary Materials

The following supporting information can be downloaded at: https://www.mdpi.com/article/10.3390/gels9020142/s1, Table S1: Least gelation Concentration (LGC) of rice varieties; Figure S1: AAC% of the selected rice varieties; Figure S2: Minimum cooking time (MCT) of rice varieties; Figure S3: Swelling ratio of retrograded rice samples (a) Diasang lahi, (b) Khaju lahi, (c) Dhusuri bao, (d) Omkar, and (e) Bili rajamudi. [34].
