# Supplementary material for: Effect of Storage Time and Temperature on Digestibility, Thermal, and Rheological Properties of Retrograded Rice
Source: Gels. 2023 Feb 8;9(2):142. doi: 10.3390/gels9020142 (PMC9957499; doi:10.3390/gels9020142)
Supplement: Supplementary file 1 [file gels-09-00142-s001.zip › gels-2160953-supplementary.pdf]

Effect of Storage Time and Temperature on Digestibility, Thermal, and Rheological Properties of Retrograded Rice

Ishita Chakraborty [1], Indira Govindaraju [1], Steffi Kunnel [1], Vishwanath Managuli [2] and Nirmal Mazumder [1,*]

[1] Department of Biophysics, Manipal School of Life Sciences, Manipal Academy of Higher Education, Manipal 576104, Karnataka, India
[2] Department of Mechanical and Industrial Engineering, Manipal Institute of Technology, Manipal Academy of Higher Education, Manipal, Karnataka, India-576104
* Correspondence: nirmaluva@gmail.com

1. Apparent amylose content

The apparent amylose content of raw rice were estimated using the iodine binding assay. 100 mg of starches were weighed and mixed with 0.36 g of NaOH in 9 mL of distilled water and 1 mL absolute ethanol and the flasks were heated in a boiling water bath for 10 min followed by cooling. Later, 1 mL acetic acid and 2 mL iodine solution were added. The volumes were made up to 100 mL and left at room temperature for 1 h. The 200 µL of the samples were loaded in 96 well plates and the optical density (OD) was then recorded at 550 nm in triplicates from which the apparent amylose contents were estimated [2].

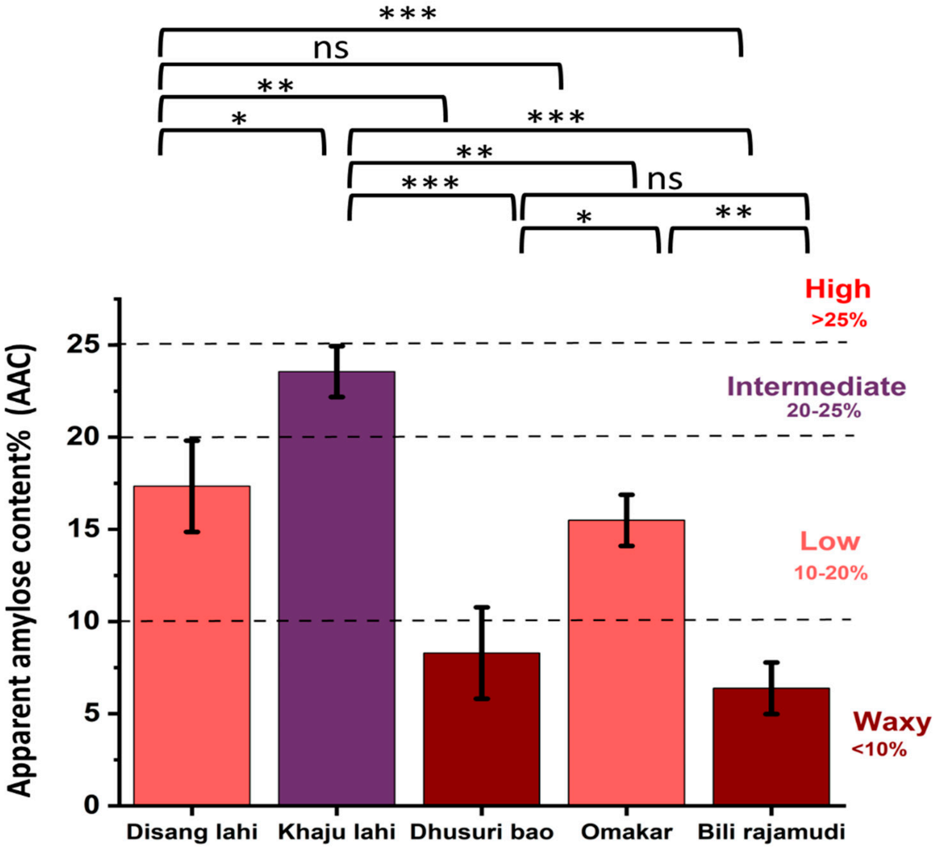

Figure S1: AAC% of the selected rice varieties

Rice varieties are grouped on the basis of their AAC% into waxy (<10% AAC), low (10-20% AC), intermediate (20–25% AC), and high (>25% AC).

2. **Least gelation Concentration (LGC)**

Least gelation concentration (LGC) was performed according to the method described in Mohamad Yazid et al., 2019 [34]. This test was done to determine the ideal starch concentration to form gel by turning test tube upside down. Suspension of starch ranging from 4-20% (w/v) was prepared in different test tubes containing 5 mL of distilled water. The starch suspension was mixed by using a vortex mixer for 2 min and heated in a water bath for 30 min at 80°C. It was then undergoing rapid cooling using running cold tap water and further cooled at 4°C for 3 h. The lowest starch concentration that form gel and did not collapse or slip with the inverted test tube was selected as the LGC. It was noted that the LGC for Dhusuri bao, Khaju lahi, and Billi rajamudi was between 8-12%. Diasang lahi shows the least LGC between 4-8%. Bili rajamudi showed the highest LGC between 12-16%.

Table S1 Least gelation Concentration (LGC) of rice varieties

**Concentration (%)**	***Dhusuri Bao***	***Khaju Lahi***	***Diasang Lahi***	***Omkar***	***Bili Rajamudi***
4%	Viscous	Viscous	Viscous	Viscous	Viscous
8%	Viscous	Viscous	Gel	Viscous	Viscous
12%	Gel	Gel	Gel	Viscous	Gel
16%	Gel	Gel	Gel	Gel	Gel
20%	Gel	Gel	Gel	Gel	Gel

3. **Minimum cooking time and swelling ratio**

To determine the minimum cooking temperature (MCT), 2 g of samples were taken in a test tube from each variety and cooked in 20 ml distilled water in a boiling water bath. The cooking time was determined by removing a few kernels at different time intervals during cooking and pressing them between two glass plates until no white core was left. The swelling ratio was determined as the fractional increase in the weight of the rice kernels due to water absorption.

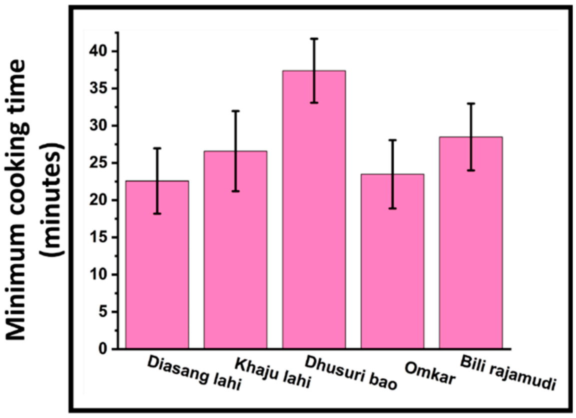

Figure S2: Minimum cooking time (MCT) of rice varieties

It was observed that Dhusuri bao exhibited the highest MCT whereas Diasang lahi showed the least MCT.

4. **Determination of swelling ratio**

The swelling ratio was determined as the increase in the weight of the rice kernels due to water absorption. 10 kernels of each rice variety were pre weighed and stirred in 20 mL of distilled water for exactly 30 min.

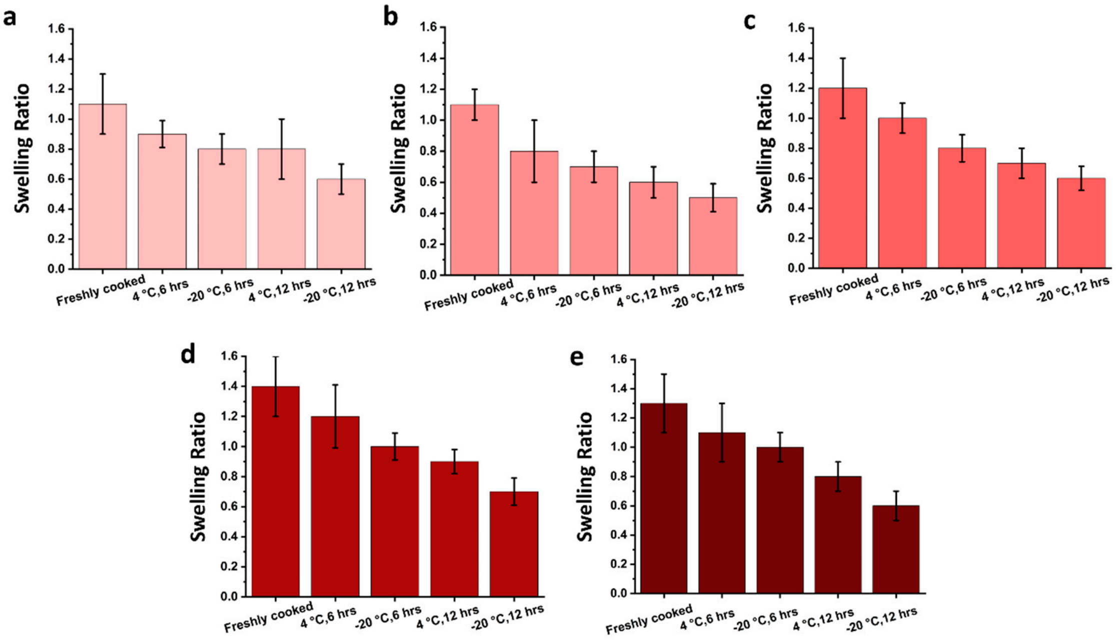

Figure S3: Swelling ratio of retrograded rice samples a) Diasang lahi, b) Khaju lahi, c) Dhusuri bao, d) Omkar, and e) Bili rajamudi

Further, it was observed that the swelling ratio decreases with increasing time and decreasing temperature of storage. The increasing RS% content in retrograded rice exhibits low water binding properties. Due to this lack of moisture, the swelling is delayed and the T_p is raised.
